# 

**Published:** 1887-09-17

**Authors:** 


The Hospital.

An Institution, Family, and Parochial Journal of Hospitals, Asylums, and all Agencies for the Care of the Sick, Criticism and News.

SATURDAY, SEPTEMBER 17th, 1887.

### Notice to Advertisers.

The scale of charges for small prepaid Advertisements—Situations Wanted, Situations Vacant, etc., etc., is as follows :—

| | s. | d. |
|---|---|---|
| One insertion of three lines | 1 | 0 |
| Three „ „ „ | 2 | 6 |
| Per line afterwards | 0 | 6 |

*A line averages eight words.*

All copy for Advertisements must be sent to A. P. Watt, 2, Paternoster Square, E.C., by first post on the Tuesday *preceding* publication.

### NOTICE TO CORRESPONDENTS.

All correspondence must be written on one side of the paper only.
We cannot undertake to return MSS. not used.
**Communications,** whether intended for insertion or for private information, must be authenticated by the names and addresses of the writers, not necessarily for publication.
**Local papers** containing reports or news paragraphs **should be marked.**
It is especially requested that early intelligence of **local events of interest,** or which it may be thought desirable to bring under notice, may be sent direct to the Editor.
Communications respecting editorial matters should be addressed to the Editor, Norfolk House, Norfolk Street, Strand, London, W.C.

### Questions and Answers.

All questions should be addressed to Mr. Howard J. Collins, Secretary, The Hospitals Association, Norfolk House, Norfolk Street, Strand, W.C.

### MASSAGE AND MEDICAL ELECTRICITY.

Nurse Lucas.—There is a "school" connected with the West End Hospital, 73, Welbeck Street, W., where nurses are taught massage and medical electricity. The fee for each course (consisting of three lectures) is £1 1s. The lectures on massage are delivered by Dr. Stretch Dowse, and on medical electricity by Dr. Tibbits. Further information can be obtained from the Lady Superintendent.—[Ed. T. H.]

### The Children's Corner.

**Fourth Quarterly Prize Competition.**
Began 2nd July, 1887 ; ends 24th Sept., 1887.

**Puzzles—Twelfth Week.**

No. 46. HISTORICAL ACROSTIC. 1. A twice-famed battle-field. 2. A famous battle of Henry V. 3. One of the Peninsular battles. 4. A battle of Henry of Navarre. 5 A battle-field where three Emperors met. Initials give the name of a town in Italy.

No. 47. MISSING VOWEL PUZZLE.

C×nsth××rs××d×n×th×br××k×rsr××r
F×rm×th×nksw×sh××ldb×n××rth×sh×r×
N×wwh×r×w××r××c×nn×tt×ll
B×t×w×sh×c××ldh××rth××nchc×p×b×ll

No. 48. BURIED ISLANDS. 1. He was borne over the ditch. 2. Frank and a man fell into the river. 3. Percy, prussic acid is a deadly poison. 4. The curlew is a fine bird.

No. 49. CONUNDRUM. What two French towns express an ill-fitting garment.

**Letters—Twelfth Week.**

Instead of giving you a letter-subject for next week, I wish you each to choose your own.

**Results of Tenth Week—Puzzles.**

No. 38. ENGLISH TOWNS ANAGRAMMATICALLY EXPRESSED. 1. Oak-ham. 2. Apple-by. 3. Cam-bridge. 4. Shef-(chef) field=Sheffield.

No. 39. DIAMOND PUZZLE.

H
d O n
m e L o n
H O L I D A Y
d o D g e
c A b
(W) Y (e)

No. 40. CHARADE. This puzzle has given rise to some amusing solutions. The correct answer is Newspaper, but I have also received shoe-horn, letter-carrier or postman, tea-spoon, firewood, etc.

No. 41. The estate was divided thus:

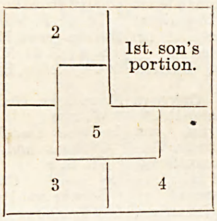

*All right*—Scrooge, Mount Edgcumbe, A. C. K., Agatha ; *Three right*—Skye, Ossian, Umslopogaas, Toby ; *Two right*—The Eda, Fern, A. G. S. McCulloch ; *One right*—Princess Ida, Poodle.

**Letters.**

The letters this week on outdoor amusements are all enthusiastic in praise of football, tennis, racquets, fives, and the less scientific game of rounders. But cricket is *par excellence* the favourite with boys and with some girls too ; while tobogganing and skating are pronounced delightful recreations for winter days. Here are a few of the letters. Toby writes :—

My favourite outdoor game is that truly English pastime, cricket. It is the national game, and there could be none better. It has gone through changes enough to spoil any ordinary game, but it has lived through all the changes, and is still the king of outdoor games. When it was first started, the bat in use was a piece of curved wood like a club ; the bowling was underhand, and beautifully pitched, and the bowlers got a splendid twist on the ball, that would dismiss many of the crack batsmen of the present day, if there were anyone who could bowl them. The bats gradually altered in shape, till they arrived at that now in use. However, at first there was no standard for the size of the blade, and consequently bats often covered half the wicket. On this the M.C.C. started a standard frame, through which every bat must pass. On one occasion a great match was to be played, and one of the players had been having a bat made for him. It was a beautiful piece of work, but it had one fault. It was wider than the wicket. The other side, seeing this, demanded that it should be passed through the frame. To accomplish this, the bat was hacked with penknives, and thus spoilt. All bats are now made of the same width, and the excellent splices put in by the makers prevent the bats from stinging your hands—a very common occurrence with unspliced bats. The bowling has gone through various changes, from the underhand twister to the roundarm, which in its turn gave place to the overarm, which is now in force, in spite of the efforts of some county bowlers, a few years ago, to change it into the ordinary shy, which would be dangerous to the batsmen, and play sad havoc with the wickets.

Ossian describes the delights of tobogganing :—

I am not what might be called keen at outdoor amusements except tobogganing, and that I really do like heartily. I could stay out a whole winter day without thinking of dinner or anything else, till my hands and my nose were frozen. A suitable day for tobogganing sends an indescribable thrill of delight through me, to be equalled only in intensity by the feeling of disgust at seeing the thaw set in. I do not think that the new-fashioned artificial tobogganing can be nearly such fun as the real. The grand stimulant of snow and frost, sliding, falling, and getting up again, together with all the seasonable wintry accessories, are what make this the king of outdoor amusements to my mind.

Ellie sends me a ballad on cricket, which is very amusing. I should be glad to know its source.

Pressure on space prevents the insertion of Socrates' interesting letter.

Three marks each :—Socrates, Toby, Ossian, Skye. Two marks each :—Princess Ida, Ellie, Mount Edgcumbe.

Answers must be addressed to the "PUZZLE EDITOR," THE HOSPITAL, Norfolk House, Norfolk Street, Strand, W.C., not later than Thursday next. The "Children's Corner" Coupon (see advertisement pages) must be enclosed.

The address and full Christian name and surname, and the age of the competitor, must be stated in all replies. A *nom de plume* may be added for the purposes of publication.

### Amusements with Profit.

### PRIZE COMPETITIONS.

**Quarterly Prize Competition.**
Began 23rd July ; ends 15th October.

**No. 17. Double Acrostic.**

My *first*, lovely goddess, comes laughing and singing,
While round her the leaves and the blossoms are clinging.
But, alas for my *second !* with short fleeting day,
He steals the bright gifts of the former away.

1. A thing very wholesome, but ah ! as you drink it,
   You show by your visage how nasty you think it.
2. A country that's famous for earthquakes and gold,
   Whose perfumes are countless, whose wealth is untold.
3. What's welcomed with joy when we're hungry and tired,
   But is most by the idle with longing desired.
4. A speech or an action, spontaneous and free,
   When our words and our actions we cannot foresee.
5. The frozen Inferno of dark Northern fable,
   Where Fenris the wolf roams round famine's bare table.
6. A monster, whose form is the arms, when combined,
   Of Old England before, and of Prussia behind.

**No. 18. Enigma.**

In helmet and corslet of steel shining bright,
Like the grim ghost in Hamlet, I'm seen in the night.
My temper's not bad, though it often takes fire;
By nature I'm rough, yet a polish acquire.
Although without wings, I can take a bold flight,
And eagle-like soar to the source of the light.
Of tobacco I nowise encourage the trade,
Yet indulge much in one thing, that from it is made.

**Answers.**

No. 13. DOUBLE ACROSTIC.

D eat H

I de A

S urrende R

C hu M

O d O

R u N

D a Y

Correct answers have been received from Ellice, Mater, Toby, Perseverance, Gretta, Condorcet.

No. 14. CHARADE.
Sea-sons = Seasons.

Correct answers have been received from Common Sense, Brownie, Condorcet, Aralc, Gretta, V. W., Toby, Mater, Ellice.

Answers must be addressed to THE PRIZE EDITOR, AMUSEMENTS WITH PROFIT, THE HOSPITAL, Norfolk House, Norfolk Street, Strand, W.C., not later than the *first* post on Friday next. The full name and address must in all cases be given, but a *nom de plume* may be added for publication. Only one side of the paper should be written on. The "Amusements with Profit" Coupon (see advertisement pages) must be enclosed with replies.

### The In- and Out-Patients' Hospital Diary.

This Diary is so arranged as to make some portion of it appear every week, and the whole in each monthly part. It has been very difficult to compile, and corrections will at all times be gratefully received. It has been suggested that similar information about the larger Provincial and Scotch Hospitals would be useful to many readers. We should be glad to hear if this is felt to be the case.

### GENERAL HOSPITALS.—*continued.*

| Hospitals and Terms of Admission. | Physicians, Surgeons, and Medical Officers, with Days and Hours of Attendance. |
|---|---|
| St. Mary's Hospital, Cambridge Place, Paddington, W. Sec., Mr. Pietro Michelli<br>In-patients are admitted by letter of recommendation. Urgent cases and accidents free at all times. Out-patients require a letter. Electrical treatment Tu. and F. 2 | *In-Physicians*, Sir Edward Sieveking, Tu. F. 1.45; Drs. Broadbent, M. Th. 1.45; Cheadle, W. 1.45, S. 9.30<br>*In-Surgeons*, Messrs. Norton, M. Th. 1.45; Owen, Tu. F. 1.45; H. Page, W. S. 1.45<br>*Out-Physicians*, Drs. D. B. Lees, W. S.; S. Phillips, M. Th.; R. Macguire, Tu. F. Hour, 1.30<br>*Out-Surgeons*, Messrs. W. Pye, M. Th.; A. J. Pepper. Tu. F.; A. Q. Silcock, W. S. Hour, 1.30 |
| St. Thomas's Hospital, Westminster Bridge Road, S.E. Steward, Mr. F. Walker<br>Accidents and emergencies free at all times. Other cases by Governor's letter, but, where unobtainable, application should be made to the Steward by letter, or personally between 10 and 4. Vaccination on W. at 11.30 | *In-Physicians*, Drs. Bristowe, Tu. F.; Stone, M. Th.; Ord, M. Th.; J. Harley, Tu. F.; Gervis, Tu. F. Hour, 2<br>*In-Surgeons*. Messrs. Sydney Jones, Tu. F.; Croft, M. Th.; Sir W. MacCormac, M. Th. Hour, 2<br>*Out-Physicians*, Drs. Payne, Tu. F.; Sharkey, M. Th.; Gulliver, W. S. Hour, 1.30<br>*Out-Surgeons*, Messrs. Clutton, Tu. F.; Anderson, W. S.; Pitts, M. Tu. Hour, 1.30 |
| Tottenham Training Hospital, Tottenham, N. Director, Dr. Laseron<br>Free. Accidents and emergencies admitted at all times | *In-Physician*. Dr. Rasch, Tu. F. 3 to 5<br>*In-Surgeons*, Drs. Lichtenberg, M. Th. 3 to 5; E. H. May, M. Th. 3 to 5<br>*Out-Surgeon*, Dr. Lloyd Smith, M. W. 11 to 2; *Men* only, W. 7 to 8 p.m. |
| University College Hospital (or North London), Gower Street, W.C. Sec., Mr. N. H. Nixon<br>Accidents and emergencies free. All other cases by Governor's letter of recommendation | *In-Physicians*, Drs. W. Fox, Tu. Th. 2, S. 9.30; Ringer, M. W. F. 1.30; Bastian, M. 2.30, W. 10, F. 2.30; F. Roberts, Tu. 1.15, Th. 1.30, S. 9.30<br>*In-Surgeons*, Messrs. B. Hill, Tu. 2.15, W. 2, F. 2.15; C. Heath, M. W. F. 2; M. Beck, Tu. Th. S. 2<br>*Out-Physicians*, Drs. Gowers, W. S.; Poore, Tu. F.; Barlow, M. Th. Hour 1.30<br>*Out-Surgeons*, Messrs. Barker, M. Th.; Godlee, Tu. F.; Horsley, W. S. Hour, 1.30 |
| West London Hospital, Hammersmith Road, W. Sec. and Supt., Mr. R. J. Gilbert<br>Accidents and emergencies free at all times. Other cases require a letter of recommendation. New cases must attend at 1.30 | *In-Physicians*, Drs. G. Rogers, D. W. Hood, F. Drewitt<br>*In-Surgeons*, Messrs. C. Keetley, F. Edwards, W. B. Clarke<br>*Out-Physicians*, Drs. Herringham, M. Th.; Ball, Tu. F.; S. Taylor, W. S. Hour, 2<br>*Out-Surgeons*, Messrs. Ballance, Tu. F.; Weiss, M. Th.; Wainewright, W. S. Hour, 2 |
| Westminster Hospital, Broad Sanctuary, S.W. Sec., Mr. S. M. Quennell<br>Accidents and urgent cases free at all times. Other cases require a letter of recommendation | *In-Physicians*, Drs. Sturges, M. Th. S.; Allchin, M. Tu. F.; Donkin, Tu. W. S. Hour, 1.30<br>*In-Surgeons*, Messrs. Cowell, M. Tu. Th.; Davy, M. Tu. F.; Macnamara, M. W. S. Hour, 1.30<br>*Out-Physicians*, Drs. De H. Hall, M. Th.; A. H. Bennett, Tu. F.; Murrell, W. S. Hour, 1.30<br>*Out-Surgeons*, Messrs. Cooke, M. Th.; Bond, Tu. F.; Barrow, W. S. Hour, 1.30 |

### II.—SPECIAL HOSPITALS.

### CANCER.

| | |
|---|---|
| Cancer Hospital, Brompton, S.W. Sec., Mr. W. H. Hughes<br>Entirely free to both in- and out-patients | *In- and Out-Surgeons*, Drs. A. Marsden, W.; H. Snow, M.; F. A. Purcell, F.; Messrs. F. B. Jessett, W.; C. Stonham, Th.; C. Jennings, Tu.; W. H. Elam, S. Hour, 2 |
| London Hospital, Whitechapel Road, E. | Daily, 1.30. *See* General Hospitals |
| Middlesex Hospital, Berners Street, W. Admission free | *In-Surgeons*, Messrs. Hulke, Lawson, and Morris<br>*Out-Surgeon*, Mr. Morris, Th. 1.30 |
| St. Saviour's Hospital, Osnaburg Street, N.W. Sec., Mr. E. Poitiers<br>By Governors' letters | *In- and Out-Physician*, Dr. A. Kennedy, 10.30 and 4 daily |

### CHILDREN'S DISEASES.

| Hospitals and Terms of Admission. | Physicians, Surgeons, and Medical Officers, with Days and Hours of Attendance. |
|---|---|
| Alexandra Hospital for Children, 18, Queen Sq., W.C. Sec., Mrs. Marsh<br>For hip disease | *Physician*, Dr. Steavenson<br>*Surgeons*, Messrs. H. Marsh Tu. F.; A. Bowlby, M. Th. Hour, 4.30<br>(No out-patients) |
| All Saints' Children's Hospital, 4, Margaret Street, W.<br>For chronic joint diseases | *Surgeon*, Mr. N. Smith<br>(No out-patients) |
| Belgrave Hosp. for Children, 77 & 79, Gloucester Street, Pimlico. Hon. Secs., Capt. Stopford, and Rev. J. Storrs<br>By letters; accidents free | *In- and Out-Physicians*, Drs. W. Hope and W. Ewart, M. Th. 9<br>*In- and Out-Surgeons*, Messrs. C. T Dent and Margerison, Tu. F. 9 |
| Charing Cross Hospital, West Strand, W.C. | *Out-Physician*, Dr. Nursby, W. S. 1.30 |
| Cheyne Hospital for Sick and Incurable Children, 46, Cheyne Walk, Chelsea, S.W. Sec., Miss D. Evans | *Physician*, Dr. G. V. Poore<br>*Surgeons*, Messrs. T. P. Bartlett and J. Macready attend alternate days in afternoons. (No out-patients) |
| East London Hospital for Children and Dispensary for Women, Glamis Road, Shadwell, E. Sec., Mr. Ashton Warner<br>By Governor's letter. Urgent cases and accidents are admitted free | *In-Physicians*, Drs. Donkin, M. Th.; Eustace Smith, T. F.; Crocker, F.<br>*In-Surgeons*, Messrs. A. Cæsar, R. W. Parker, W. F.<br>*Out-Physicians*, Drs. Crocker, M.; Dawson Williams, Tu. Th.; Coutts, W. F. Hours, 1 and 3<br>*Out-Surgeon*, Mr. Dunn, Tu. F. 1, 3 |
| Evelina Hospital for Sick Children, Southwark Bridge Road, S.E. Sec., Mr. T. S. Chapman.<br>Free, but Governor's letter takes precedence. In-patients (boys) between 2 and 10, (girls) between 2 and 12 | *In-Physicians*, Drs. F. Taylor and J. Goodhart<br>*In-Surgeons*, Messrs. H. G. Howse and R. C. Lucas<br>*Out-Physicians*, Drs. N. Tirard, M. Th. 9; F. Willcocks, Tu. F. 9<br>*Out-Surgeons*, Messrs. C. J. Symonds, S. 9; G. H. Makins, W. 9 |
| German Hospital, Dalston Lane, Dalston, E. | *In-Physician*, Dr. Rasch, M. Th. 9 |
| Great Northern Central Hospital, Caledonian Rd. N. | *Physicians*, Drs. Murray and Barnes, W. 2.30 |
| Grosvenor Hospital for Women and Children, Vincent Sq., S.W. Hon. Secs., Mr. A. J. Ram and Miss Day<br>By letter or payment | *In- and Out-Physicians*, Drs. R. Gibbons and Steavenson, Tu. W. Th. S. 2<br>*In- and Out-Surgeons*, Messrs. Smythe and Parker, Tu. W. Th. S. 2 |
| Hospital for Sick Children, 48 and 49, Great Ormond St., W.C. Sec., Mr. S. Whitford<br>By Governors' letters. If without letter, the case of the applicant is investigated<br>In-patients between 2 and 12; Out-patients under 12 | *In-Physicians*, Drs. W. Cheadle, Tu. F.; Sturges, W. S.; Barlow, M. Th. Hours, 9 to 11<br>*In-Surgeons*, Messrs. H. Marsh, W. S.; E. Owen, W. S. Hours, 9 to 11<br>*Out-Physicians*, Drs. R. J. Lee and Abercrombie, M. Th.; Lubbock and Money, Tu. F. Hours, 8.30 to 10<br>*Out-Surgeons*, Messrs. Morgan and Pitts, W. S. 8.30 to 10 |
| King's College Hospital, Portugal Street, W.C. | *In- and Out-Physician*, Dr. T. C. Hayes, W. F. 1 |
| Middlesex Hospital, Berners Street, W.<br>Free. Children under 3 | *Out-Physician*, Dr. Duncan, W. S. 1.30 |
| North Eastern Hospital for Children, Hackney Road, E. Sec., Mr. A. Nixon<br>By free ticket or small payment | *In- and Out-Physicians*, Drs. W. Cayley, F. Turner, C. Semple, and W. Pasteur, M. W. Th. S. 1.30<br>*In- and Out-Surgeons*, Messrs. Waren Tay and Pollard, M. 1.30, F. 9 |
| Paddington Green Children's Hosp., Paddington Green. Sec., Mr. W. H. Pearce<br>Admission free; boys under 12; girls under 14 | *In- and Out-Physicians*, Drs. T. Lauder Brunton, Ogilvie, Tu. F.; G. Laycock, M. Th.; S. Phillips, W. S. Hour, 9.15<br>*In- and Out-Surgeons*, Messrs. W. W. Cheyne, M., S. Boyd, F. Hour, 9.15 |
| Royal Hospital for Children and Women, Waterloo Bridge Road, S.E. Sec., Mr. R. G. Kestin<br>Out-Patients pay 1*d.* on each attendance. Boys not admissible, as out-patients, over 12, or, as in-patients, over 8 | *In-Physicians*, Drs. Park, M. Th.; Gulliver, Tu. F.; Price, W. S. Hour, 2<br>*In-Surgeon*, Mr. W. H. A. Jacobson, M. Th. Hour, 2<br>*Out-Physicians*, Drs. Park, M. Th.; Dakin, W. F.; Hadden, Tu. S. Hour, 2<br>*Out-Surgeon*, Mr. E. O. Day, W. 2 |
| St. Mary's Hospital, Cambridge Place, Paddington | *Out-Physician*, Dr. M. Handfield Jones, M. Th. 1.30 |
| St. Thomas's Hospital, Westminster Bridge Road, S.E | *Out-Physician*, Dr. Cory, W. S. 1.30 |

